# The effects of diet on the physical characteristics of feces and fiber digestibility in healthy horses

**DOI:** 10.1093/jas/skag255

**Published:** 2026-08-13

**Authors:** Ashley Starrett, Jhones O Sarturi, Jessica L Leatherwood, Morgan K Chaffin, Swagata Mukhopadhyay, Rachel Pilla, Carolyn E Arnold

**Affiliations:** School of Veterinary Medicine, Texas Tech University, Amarillo, TX 79106, United States; Department of Animal and Food Sciences, Texas Tech University, Lubbock, TX 79409, United States; Department of Animal Science, Tarleton State University, Stephenville, TX 76402, United States; School of Veterinary Medicine, Texas Tech University, Amarillo, TX 79106, United States; Department of Animal and Food Sciences, Texas Tech University, Lubbock, TX 79409, United States; Gastrointestinal Laboratory, School of Veterinary Medicine and Biomedical Sciences, Texas A&M University, College Station, TX 77843, United States; Department of Veterinary Medicine and Animal Sciences, University of Milan, Lodi 26900, Italy; School of Veterinary Medicine, Texas Tech University, Amarillo, TX 79106, United States

**Keywords:** colic, diet, digestibility, equine, fecal particle size, fiber

## Abstract

Diet is a known risk factor for colic, but few studies have explored how diet affects fecal characteristics which are often altered in horses with colic. This prospective cross-over study evaluated the effects of two common equine diets on fecal characteristics and fiber digestibility in healthy adult horses. We hypothesized that horses offered a complete pelleted diet would exhibit increased fecal dry matter (DM) and decreased fecal particle size compared to those offered a forage-based diet. Twenty horses at Texas Tech University School of Veterinary Medicine served as subjects in the study. Horses were offered two diets following a 3-d transition and 10-d dietary adaptation period, during which hay was provided ad libitum for horses in the hay plus supplementation group. During the study periods, diets consisted of either: coastal Bermudagrass hay offered at 2% body weight (BW) with a commercially available complete pelleted feed at 0.7% BW, or the same complete pelleted feed offered at 1.35% of BW. Rectal grab samples were collected twice daily for 4 d, dried for 96 h at 55°C, and analyzed for fiber content and particle size. Data were analyzed using two-way ANOVA in R Studio and the GLIMMIX procedure of SAS, with treatment as a fixed effect and period and animal as random effects. Significance was set at *P *< 0.05. Horses offered the complete pelleted feed had greater amounts of mean fecal particles retained in sieve 1.70 mm (*P *= 0.02), 2.36 mm, 180 µM, and the collection pan (*P *< 0.01) compared to horses offered the forage-based diet. Horses on the forage-based diet had elevated neutral detergent fiber (NDF) and fecal gross energy (GE) compared to those on the complete pelleted feed (*P *< 0.01), whereas horses offered the complete pelleted feed had greater ash (*P *< 0.01) and acid detergent fiber (ADF; *P *= 0.04) in fecal matter. There was a tendency for higher lignin in horses offered the complete pelleted feed (*P *= 0.08). Horses consuming hay with supplementation diet showed greater intake (including DM, organic matter [OM], NDF, ADF, hemicellulose (HEM), GE, and digestible energy [DE]), fecal output OM and HEM, along with a greater apparent total tract digestibility (DM, ADF, NDF [*P *< 0.01], and HEM [*P *= 0.04]). These findings demonstrate that diet influences fecal particle size, fiber fraction, and digestibility in healthy horses, factors that may have implications for gastrointestinal health and colic risk.

## Graphical abstract

**Graphical Abstract:**
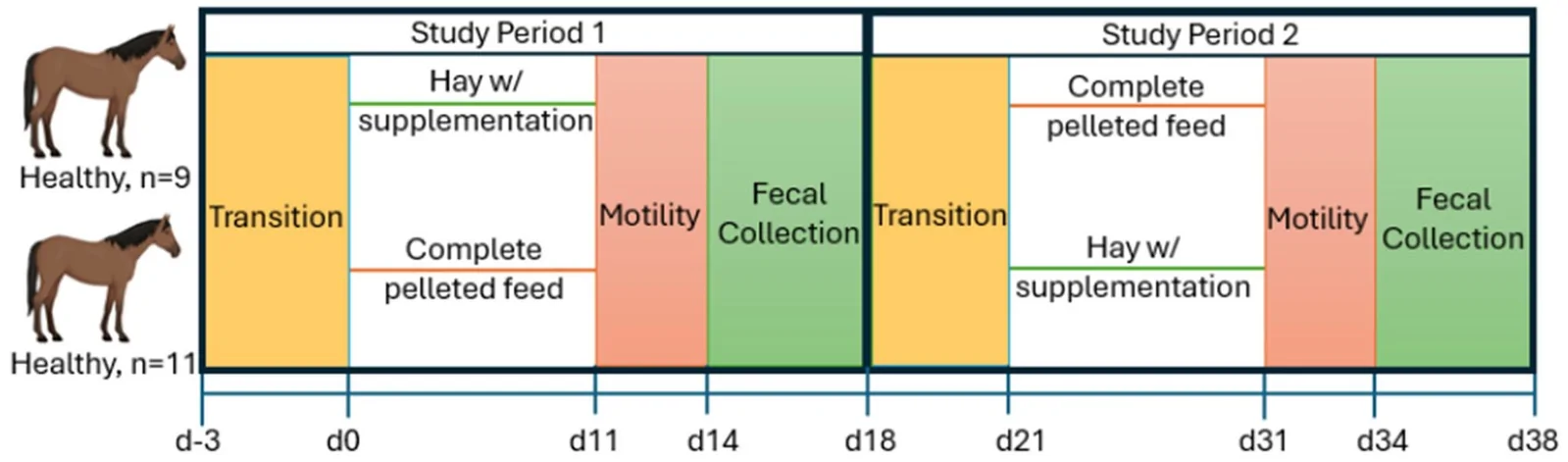

## Teaser

Can your horse’s diet influence characteristics of their manure? A recent study from Texas Tech University School of Veterinary Medicine explores how forage-based vs. pelleted diets affect fecal characteristics and fiber digestibility in healthy horses. The findings reveal significant differences in fecal particle size and fiber digestibility, key insights that could help inform feeding strategies to support optimal equine digestive health.

## Introduction

Despite advancements in equine husbandry and veterinary care, colic remains the primary cause of morbidity among horses worldwide (Curtis et al. 2019). The horses’ gastrointestinal anatomy and reliance upon hindgut microbial fermentation to utilize dietary fiber have been identified as contributing factors in the development of colic. Because fiber digestion relies on a stable hindgut environment and microbial population, dietary composition and feeding management can substantially influence gastrointestinal function. Not surprisingly, epidemiological data have consistently attributed diet, the substrate of digestion, and feeding practices as risk factors for colic. Studies have associated several dietary factors as increasing the risk of colic, including hay type (Cohen et al. 1995; Cohen 2003), frequent changes in hay source (Tinker et al. 1997; Cohen et al. 1999; Hudson et al. 2001), feeding hay from round bales vs. flakes (Hudson et al. 2001), and the amount of concentrate offered (Hudson et al. 2001). However, few studies have examined how diet impacts fecal characteristics and digestibility, both of which are closely related to gastrointestinal function. Fecal particle size may serve as a practical, non-invasive indicator of physical fiber processing and gastrointestinal function. Fecal dry matter (DM) content provides additional descriptive information regarding digesta water content and fecal consistency, which may also reflect changes in gastrointestinal function. Deviations in these fecal parameters could signal subclinical dysfunction, such as impaired fiber fermentation or altered gut motility, that may precede clinical signs of colic (Grimm et al. 2017).

Gonçalves et al. (2006) reported that fecal characteristics were altered in horses with colic compared to healthy horses. Differences in fecal parameters were noted in horses with obstructive vs. non-obstructive lesions, and in the anatomical segment of the gastrointestinal tract affected by colic (foregut vs. hindgut). Horses with obstructive colic exhibited significant alterations in fecal consistency, color, quantity, time since last defecation, and the presence of feed particles. These changes were attributed to increased digesta retention time and enhanced water absorption associated with intestinal obstruction, highlighting a potential link between fecal characteristics and gastrointestinal transit. This finding underscores the importance of monitoring fecal characteristics as a potential indicator of gastrointestinal disease, particularly in conditions involving impaired digesta movement. However, the effects of different diets on the fecal characteristics in healthy horses are yet unknown. To address this gap, the present study investigated how feeding programs, a forage-based diet and complete pelleted feed influence the fecal characteristics and fiber digestibility in healthy adult horses. Complete pelleted feeds are designed as forage-related diets, supplying both the fiber and nutrients necessary to replace traditional hay-based rations when fed according to the manufacture’s recommendations. The authors hypothesize that horses offered a complete pelleted diet would exhibit greater fiber digestibility and less fiber fractions compared with those offered a forage-based diet.

## Materials and methods

The following experimental procedures were conducted in accordance with the Texas Tech Institutional Animal Care and Use Committee (IACUC 2023-1454).

### Animals, experimental design, and facility

Twenty adult horses (19 American Quarter Horses and one Thoroughbred; median 16.7 yr, range 5–26; 15 mares and 5 geldings) at the Texas Tech University School of Veterinary Medicine served as subjects in a prospective, cross-over study. Horses were randomly assigned to treatment sequence based on pre-assigned turnout groups balanced by body weight (BW) and sex (Table S1). The horses had been acclimated to the herd for approximately 3 yr and had known health histories, deworming, vaccination, dental care, and no prior history of colic or other gastrointestinal disease. No severe malocclusions were observed in any of the horses’ oral cavities. BWs were taken using a digital platform scale (Brecknell, Fairmont, MN), and body condition scores (BCS) were assessed by one equine nutritionist at three-time points: prior to the start of the study, at the cross-over point, and at the conclusion of the study. A nine-point scale developed by Henneke et al. (1983) was used to assign scores, ranging from BCS one (*very poor*) to BCS nine (*extremely fat*).

Each experimental period consisted of a 3-d dietary transition where one-third of the new diet was added each day, a 10-day dietary adaptation phase (Study Day 1–10), 3 d in which gastrointestinal motility was measured for an unrelated study (Study Day 11–13), a 4-d fecal, diet, and refusal collection phase (Study Day 14–17). During the dietary transition and adaptation phases, the horses were offered complete pelleted feed in individual 3.7 × 3.7 m stalls with rubber mats connected to a 7.0 × 3.7 m soil runs. Horses were allowed 2 h every morning and evening to eat their completed pelleted feed from a plastic bucket in their individual stalls with runs and unlimited turnout in groups on 0.405 ha dirt lots between feeding times. Horses in the hay group were offered hay ad libitum in a hay manger for the first 10 d of the study. On study day 11–17, horses were kept in individual stalls and runs 24 h per day to facilitate fecal collection, diets, and refusals. During this time hay was offered in individual barrels in horses stalls and refusals collected 2 h post morning and evening feedings to prevent contamination.

### Diets, treatments, and feeding strategy

Both study diets were designed to meet or exceed NRC requirements for mature horses at maintenance (NRC 2007). The two diets consisted of (1) coastal Bermudagrass (*Cynodon dactylon*) hay offered at 2% BW with a commercially available complete pelleted feed (Equine Senior, Purina, Gray Summit, Missouri) offered at 0.7% BW (hay with supplementation) and (2) the same complete pelleted feed offered at 1.35% BW per manufacturer. Fresh water was provided ad libitum throughout the study.

### Chemical composition

#### Feed analysis

Hay samples were collected from multiple random square bales offered to horses in a dry lot on day zero of each study period. Samples of the complete pelleted feed were collected from 50-lb bags on the same day. Both hay and concentrate were analyzed for acid detergent fiber (ADF), neutral detergent fiber (NDF), lignin, crude protein (CP), and ash (ServiTech Laboratories, Amarillo, TX). Feedstuffs were initially dried at 60°C for 16 h, ground to pass a 1 mm screen in a Retsch SR 300 Mill (Retsch GmbH, Haan, Germany) and subsamples subjected to a second drying at 105°C for 3 h. The ADF and NDF were analyzed using Ankom Technology filter bag techniques, as described by van Soest et al. (1991) and with added thermostable α-amylase and sodium sulfite, with lignin determined from ADF and NDF residues. All fiber fractions were corrected for the residual ash content (AOAC 2005). Ash was determined according to Method 942.05 (AOAC 2012). CP was analyzed according to Method 990.03 (Thiex 2013). Gross energy (GE) for diets was determined using an IKA C2000 or C3000 Calorimeter System (Dairy One, Ithaca, NY).

#### Fecal collection

On study day 14–17, horses were housed individually in stalls with attached runs for 24 h/d to facilitate sampling during a 3-h advancing collection window. Fecal samples (∼200 g/d per horse) were obtained via natural defecation when possible or rectal grab when necessary to ensure adequate sample. Fecal samples were thoroughly homogenized by manual mixing prior to analyzes to ensure representative distribution of material across each assay.

#### Fecal analysis

Fecal samples were analyzed for DM, ADF, NDF, lignin, and ash at ServiTech Laboratories (Amarillo, TX). The fecal samples were thawed and dried in a VWR 1390FM forced-air oven (VWR International, Radnor, PA) at 55°C for 96 h as described by Silva et al. (2015). Dried fecal samples were ground in a Retsch SR 300 Mill with a 1 mm screen (Retsch GmbH, Haan, Germany). Analysis of ADF and NDF were determined using the filter bag technique (Ankom, Macedon, NY) based on the methods of Van Soest et al. (1991), as described above. Lignin was calculated from ADF and NDF residues. GE for feces was determined using an IKA C2000 or C3000 Calorimeter System (Dairy One, Ithaca, NY).

### Apparent total tract nutrient digestibility

The digestibility assessment was conducted from d 14 to 17 of each period. Diet and refusal samples were collected 2 h post feeding time, while feces (∼200 g, as-is basis) were individually collected (natural elimination or rectal grab) every 12 h on an advancing 3-h schedule over 72 h to account for diurnal variation. Samples were immediately aliquoted for each assay and stored at −20°C.

#### Titanium

Titanium dioxide (TiO_2_; Sigma-Aldrich, Milwaukee, WI) served as an external marker for the estimation of fecal output. It was top-dressed on the complete pelleted feed at 5 g twice daily for the last 14 d leading up to and including fecal collection days for each phase of the study (Study Day 4–17). The total feed input was measured, and total fecal output was determined for each horse. The apparent total tract digestibility for DM, organic matter (OM), ADF, NDF, hemicellulose (HEM), and digestible energy (DE) was calculated as follows:


$$\mathrm{Apparent}\;\mathrm{nutrient}\;\mathrm{digestibility},\;\%=100-100\;\times \;[\frac{\mathrm{conc}.\;\mathrm{of}\;\mathrm{TiO}2\;\mathrm{in}\;\mathrm{feed}}{\mathrm{conc}.\;\mathrm{of}\;\mathrm{TiO}2\;\mathrm{in}\;\mathrm{feces}}\times \frac{\mathrm{conc}.\;\mathrm{of}\;\mathrm{nutrient}\;\mathrm{in}\;\mathrm{feces}}{\mathrm{conc}.\;\mathrm{of}\;\mathrm{nutrient}\;\mathrm{in}\;\mathrm{feed}}]$$

Titanium was measured by using dried ground feces and a portable X-ray fluorescence (PXRF) spectrometry (Vanta, Olympus, Waltham, MA, USA; Hoffmann et al. 2020). A GeoChem mode was selected for scanning (Weindorf and Chakraborty 2020) using a 10 kV, 40 kV, and 50 kV beam for 30 s each. Samples were prepared using polyethylene PXRF cups and covered with 4 μM polypropylene film during scanning to protect the PXRF window from the dust. The PXRF was calibrated using an alloy coin provided by the manufacturer (316, 96 Olympus, Waltham, MA, USA). Additionally, two National Institute of Standards and Technology certified standards, 2710a (Montana I soil) and 2711a (Montana II soil), were analyzed. Correction factors were calculated as the ratio of the certified value of an element in the standard to its corresponding PXRF value.

### Physical characteristics of feces

#### Fecal particle size

Fecal particle size was assessed using dried, unground fecal samples and a Sieve Shaker (Model SS-8R; Gilson Company, Inc., Worthington, OH). Each sample was gently broken apart by hand to separate clumps before sieving. Samples were placed in a stack of seven sieves with decreasing pore sizes (4.75 mm, 2.36 mm, 1.70 mm, 1.18 mm, 1.00 mm, 180 µM) and a collection pan at the base. The dry sieving process mechanically agitated samples for 2 min to separate particles by size, which were retained on the corresponding sieves. Between samples, sieves were thoroughly cleaned to prevent cross-contamination. The retained material on each sieve and the collection pan was weighed to determine the mean of fecal particles within each size fraction. The distribution of fecal particle sizes among pans was calculated.

### Statistical analysis

Physical characteristics of feces and the chemical composition of feedstuff and fecal fiber were analyzed using R Studio (RStudio Team 2023; Version 2023.06.2 + 561). Data sets were analyzed for normality with a Shapiro-Wilk test and Levene’s test to check for homogeneity prior to using a two-way ANOVA. *P*-values of less than 0.05 were considered significant.

Digestibility data of horses were analyzed using the GLIMMIX procedure of SAS (SAS Inst., Inc., Cary, NC) in a 2 × 2 prospective cross-over design (two periods and two treatments) with the horses served as the experimental unit. For analysis of intake and apparent total tract digestibility, the model included the fixed effects of treatment and random effects of period and horse within period. The general degrees-of-freedom procedure Kenward and Rogers (1997) was used to adjust for any bias on standard errors caused by multiple terms in the random statement. The differences were considered significant at *P *< 0.05 and meaningful tendencies discussed when 0.05 < *P *≤ 0.15.

## Results

### Chemical composition

The analyzed chemical composition of the feedstuffs is presented in Table 1. Differences in nutrient composition between the complete pelleted feed and coastal Bermudagrass hay were consistent with the expected composition of these feedstuffs, including greater NDF concentrations in the hay and greater CP and DE concentrations in the pelleted feed. The chemical composition of feces was affected for NDF, GE, ash and a tendency for higher lignin between treatment groups (Table 1). Horses on the forage-based diet had elevated fecal NDF and GE compared to those on the complete pelleted feed (*P *< 0.01), whereas horses offered the complete pelleted feed had greater fecal ash (*P *< 0.01) and ADF (*P *= 0.04). There was a tendency for higher fecal lignin in horses offered the complete pelleted feed (*P *= 0.08). No differences were observed in fecal DM between the diets (*P *= 0.26).

**Table 1:** Nutrient composition of feedstuff and feces for healthy horses offered two common equine diets.

Nutrient^1^	*n*	Treatment (median)		SD	*P*-value
		**Hay^2^**	**Complete pelleted feed^3^**		
**Feedstuff**					
** CP, %**	2	14.35	16.00	1.17	0.33
** ADF, %**	2	33.10	25.55	5.34	0.33
** NDF, %**	2	66.00	38.70	19.29	0.33
** Lignin, %**	2	6.35	6.30	0.04	0.99
** Ash, %**	2	7.75	9.40	1.17	0.33
** GE, Mcal/kg**	2	4.41	4.49	0.06	0.33
** DE, Mcal/kg**	2	2.89	2.98	0.06	0.33

		**Hay with supplementation** ^4^	**Complete pelleted feed**		

**Feces**					
** DM, %**	20	96.77	95.68	1.38	0.26
** ADF, %**	20	44.05	48.55	4.71	0.04
** NDF, %**	20	69.00	59.50	2.64	*<*0.01
** Lignin, %**	20	10.30	12.70	2.06	0.08
** Ash, %**	20	13.00	27.75	3.08	*<*0.01
** GE, Mcal/kg**	20	4.50	3.92	0.23	*<*0.01
** DE, Mcal/kg**	20	3.81	3.79	0.09	0.28

1 Values presented on a 100% DM basis.

2 Coastal Bermudagrass (*Cynodon dactylon*) hay was offered ad libitum to all horses.

3 Complete Pelleted feed (Equine Senior, Purina, Gray Summit, Missouri) offered at 1.35% BW per manufacturer.

4 Coastal Bermudagrass (*Cynodon dactylon*) hay offered at 2% BW with a commercially available complete pelleted feed offered at 0.7% BW.

### Intake, total fecal output, and digestibility

Intake, total fecal output, and digestibility of healthy horses offered two common equine diets are summarized in Table 2. DM intake, OM intake, NDF intake, ADF intake, HEM intake, GE intake, and DE intake were greater in horses offered the hay with supplementation compared to those offered the complete pelleted feed (*P *< 0.01).

**Table 2:** Intake, total fecal output and digestibility of healthy horses offered two common equine diets.

Nutrient	Treatment (median)		SEM	*P*-value
	**Hay with supplementation^1^**	**Complete pelleted feed^2^**		
**Intake**				
** DM, kg/d**	8.73	6.12	0.29	<0.01
** OM, kg/d**	8.00	5.55	0.27	<0.01
** NDF, kg/d**	4.89	2.36	0.20	<0.01
** ADF, kg/d**	2.65	1.56	0.10	<0.01
** HEM, kg/d**	2.24	0.80	0.11	<0.01
** GE, Mcal/d**	38.70	27.49	1.27	<0.01
** DE, Mcal/d**	33.25	23.23	1.12	<0.01
**Total fecal output**				
** DM, kg/d**	1.18	1.07	0.06	0.10
** OM, g/day**	1019.98	745.36	54.59	<0.01
** NDF, g/day**	845.18	667.06	48.11	0.15
** ADF, g/day**	528.62	521.85	40.24	0.87
** HEM, g/day**	316.56	145.21	17.76	<0.01
** Fecal energy loss (% GE intake)**	13.50	15.50	0.54	<0.01
**Apparent total tract digestibility**				
** DM, %**	86.47	82.51	0.63	<0.01
** OM, %**	87.24	86.56	0.53	0.21
** NDF, %**	82.61	71.77	1.07	<0.01
** ADF, %**	79.80	66.57	1.77	<0.01
** HEM, %**	85.86	81.82	1.85	0.04
** DE, Mcal/kg**	3.81	3.79	0.03	0.37

1 Coastal Bermudagrass (*Cynodon dactylon*) hay offered at 2% BW with a commercially available complete pelleted feed (Equine Senior, Purina, Gray Summit, Missouri) offered at 0.7% BW.

2 Complete Pelleted feed offered at 1.35% BW per manufacturer.

Estimated total fecal output of OM, HEM, and fecal energy loss (*P *< 0.01) were greater in horses offered hay with supplementation compared to the complete pelleted feed. A higher tendency was observed in fecal DM (*P *= 0.10) and NDF (*P *= 0.15) for horses offered hay with supplementation, and no difference was observed in ADF (*P *= 0.87) between the two diets.

Apparent total tract digestibility of DM, NDF, ADF (*P *< 0.01), and HEM (*P *= 0.04) were greater in horses consuming hay with supplementation diet compared to those on the complete pelleted feed. However, there were no differences in OM (*P *= 0.21) or dietary DE (*P *= 0.37) between diets.

### Physical characteristics of feces

The physical characteristics of feces are summarized in Table 3. Horses offered the complete pelleted feed had more mean particles retained in the 2.36 mm sieve, 180 µM sieve (*P *< 0.01), 1.70 mm sieve (*P *< 0.02), and the collection pan (*P *< 0.01) compared to those offered hay with supplementation.

**Table 3:** Mean fecal particles retained in each sieve size for healthy horses offered two common equine diets.

Sieve pore size	Treatment (mean, g)		SEM	*P*-value
	**Hay with supplementation^1^**	**Complete pelleted feed^2^**		
**4.75 mm**	0.20	0.23	0.02	0.23
**2.36 mm**	2.62	4.26	0.32	<0.01
**1.70 mm**	2.33	3.38	0.18	0.02
**1.18 mm**	4.62	4.88	0.32	0.56
**1.00 mm**	3.10	3.07	0.17	0.90
**180 µM**	21.43	35.82	1.20	<0.01
**Collection Pan**	2.30	4.13	0.13	<0.01

1 Coastal Bermudagrass (*Cynodon dactylon*) hay offered at 2% BW with a commercially available complete pelleted feed (Equine Senior, Purina, Gray Summit, Missouri) offered at 0.7% BW.

2 Complete Pelleted feed offered at 1.35% BW per manufacturer.

## Discussion

This study demonstrates that diet composition and physical form influence mean fecal particle size and fiber digestibility in healthy horses. Collectively, the findings did not fully support the original hypothesis, as horses offered the hay with supplementation diet exhibited greater apparent total tract digestibility of fiber fractions, while horses consuming the complete pelleted feed produced a different mean fecal particle size and greater ash, ADF and a trend for lignin concentrations in fecal matter. These results highlight that gastrointestinal responses to diet are driven by a combination of nutrient composition, physical structure, and feeding management rather than nutrient content alone.

A primary finding of this study was that the greater dry matter and nutrient intake observed in horses receiving hay with supplementation was a direct consequence of the experimental feeding design, as this group received a greater total daily allotment (2.7% BW) compared with horses fed the complete pelleted diet (1.35% BW). Therefore, intake differences should be interpreted as imposed dietary conditions rather than voluntary feed intake behavior. Despite this, differences in fecal characteristics and digestibility indicate that diet form and composition exerted independent effects on gastrointestinal processing beyond intake alone.

Differences in mean fecal particle size likely reflect the inherent properties of each diet including fiber composition, physical processing, chewing behavior, and digesta passage rate (although not measured). Horses consuming long-stem forage spend more time chewing and typically produce greater mechanical breakdown prior to ingestion, whereas pelleted feeds require minimal mastication and may reduce salivary production and buffering capacity (data not measured; Preston 2016; Fernandes et al. 2022; Martuzzi et al. 2024). In addition, the finely processed physical form and smaller particle size of pelleted feeds may accelerate gastrointestinal transit and reduce retention time for microbial fermentation in the hindgut, although gastrointestinal transit time was not measured in the present study. These combined effects may have contributed to the altered mean fecal particle size observed in horses fed the complete pelleted diet.

In contrast, horses fed hay with supplementation exhibited greater apparent total tract digestibility of DM, NDF, ADF, and HEM. These values were greater than those reported in previous studies evaluating grass hay-based diets (Frape 2010), which may reflect differences in diet composition and physical form between studies. Although forage maturity, plant composition, and lignification are known to influence fiber degradability, these variables were not directly measured in the present study. Inclusion of a commercial concentrate may also have altered hindgut fermentation dynamics and improved overall nutrient utilization. Additionally, methodological differences in digestibility estimation and variation in study population further limit direct comparison among studies reported in the literature.

An unexpected finding was reduced ADF digestibility observed in horses consuming the complete pelleted feed despite its processed physical form. While feed processing is generally expected to enhance digestibility by increasing surface area and reducing particle size, ADF digestibility is primarily governed by cellulose and lignin fractions, which are resistant to degradation regardless of processing. The pelleted feed used in this study contained ground soybean hulls, wheat middlings, dehydrated alfalfa meal, and beet pulp, resulting in an ADF max concentration of 23.0%. Although these ingredients contribute to dietary fiber, their cell wall composition varies in fermentability, with some (beet pulp and soybean hulls) being highly fermentable despite contributing to total fiber fractions. Therefore, ADF content alone does not directly reflect fiber degradability. Furthermore, increased passage rate associated with finely processed diets may have limited microbial exposure time in the hindgut, reducing fiber fermentation efficiency. Thus, physical processing alone does not necessarily improve digestion of structurally complex fiber fractions.

However, fecal fiber fractions should be interpreted cautiously, as they are strongly influenced by diet composition and do not independently reflect digestive efficiency. Previous studies have demonstrated that horses with large colon impaction have been shown to exhibit smaller fecal particle size, particularly in the presence of concurrent dental pathology that may impair effective mastication and initial particle reduction (Gunnarsdottir et al. 2014). More recent studies have further reported differences in fecal DM concentration and a redistribution of fecal particle size fractions in horses with colic compared with healthy controls, indicating altered physical processing of fiber across the gastrointestinal tract (Müller 2024). Although the present study was conducted in healthy horses, the observed diet-associated differences in fecal characteristics may provide mechanistic insight into how feeding practices could influence gastrointestinal function and potentially contribute to colic risk.

It is important to note that the observed differences in fecal characteristics and digestibility are not only solely attributable to nutrient composition but also reflect differences in physical form, feeding rate, and gastrointestinal transit dynamics. These findings are particularly relevant given the increasing use of complete pelleted feeds in performance, geriatric, and management-restricted feeding systems, where reduced chewing time and altered fiber processing may have downstream effects on hindgut function. A limitation of this study was that feeding behavior, chewing activity, and water consumption, all of which may further influence fecal output and mean fecal particle size was not measured. The relatively wide age range of horses included in the study may also have contributed to biological variation in digestive responses.

In conclusion, diet clearly impacts fecal particle size and fiber digestibility in healthy horses. The hay with supplementation diet resulted in a shift toward smaller fecal particles retained in sieves and greater DM, ADF, NDF, and HEM digestibility, whereas the complete pelleted feed had significantly more ash, ADF and retained a larger number of fecal particles. These findings emphasize that both the composition and physical form of the diet influence digestive efficiency, providing a foundation for future studies linking diet, fecal parameters, and gastrointestinal health outcomes. Such knowledge may ultimately inform feeding strategies that support optimal gut health and reduce colic risk in horses.

### Implications

Differences in diet composition and physical form may influence fiber breakdown and gastrointestinal transit, potentially contributing to variation in mean fecal particle size. The present findings suggest that pelleted and forage-based diets can produce measurable differences in fecal characteristics, which may reflect differences in mastication and fiber processing within the gastrointestinal tract. Monitoring fecal particle size may therefore provide a practical, non-invasive approach to assessing physical fiber processing within the gastrointestinal tract. In contrast, fecal fiber fractions should be interpreted in the context of diet composition and digestibility and are less informative as standalone indicators of digestive efficiency. Because manure output and consistency are routinely evaluated in horses with gastrointestinal complaints, veterinarians and owners should be aware that diet composition itself can significantly influence fecal particle size and characteristics.

## Supplementary Material

skag255_Supplementary_Data

## Abbreviations

ADF: acid detergent fiber
BCS: body condition score
BW: body weight
CP: crude protein
DM: dry matter
DE: digestible energy
GE: gross energy
HEM: hemicellulose
NDF: neutral detergent fiber
OM: organic matter
PXRF: portable x-ray fluorescence spectrometry
SAS: Statistical Analysis Software

## References

[skag255-B1] Association of Official Analytical Chemists (AOAC). 2005. Official method of analysis. 18th ed. AOAC Int.

[skag255-B2] Association of Official Analytical Chemists (AOAC). 2012. Official method of analysis. 19th ed. AOAC Int.

[skag255-B3] Ball DM , Hoveland CS, Lacefield GD. 2007. Southern forages. 4th ed. International Plant Nutrition Institute (IPNI).

[skag255-B4] Cohen ND. 2003. The John Hickman memorial lecture: colic by numbers. Equine Vet J. 35:343–349. 10.2746/042516403776014244 12880001

[skag255-B5] Cohen ND , Gibbs PG, Woods AM. 1999. Dietary and other management factors associated with colic in horses. J Am Vet Med Assoc. 215:53–60. 10.2460/javma.1999.215.01.53 10397066

[skag255-B6] Cohen ND , Matejka PL, Honnas CM, Hooper RN. 1995. Case-Control Study of the Association between various management factors and development of colic in horses. J Am Vet Med Assoc. 206:667–673. 10.2460/javma.1995.206.05.667 7744689

[skag255-B7] Fernandes KA et al 2022. Comparison of gastrointestinal transit times in stabled Thoroughbred horses fed freshly cut pasture and three conserved forage-based diets. Anim Prod Sci. 62:1192–1202.

[skag255-B9] Frape D. 2010. Equine nutrition and feeding. John Wiley & Sons.

[skag255-B10] Gonçalves S , Leblond A, Drogoul C, Julliand V. 2006. Using feces characteristics as acriterion for the diagnosis of colic in the horse: a clinical review of 207 cases. Revue Méd Vêt. 157:3–10.

[skag255-B11] Grimm P , Philippeau C, Julliand V. 2017. Faecal parameters as biomarkers of the equine hindgut microbial ecosystem under dietary change. Animal. 11:1136–1145. 28065211 10.1017/S1751731116002779

[skag255-B12] Gunnarsdottir H et al 2014. Hospital-based study of dental pathology and faecal particle size distribution in horses with large colon impaction. Vet J. 202:153–156. 10.1016/j.tvjl.2014.07.013 25135337

[skag255-B13] Henneke DR , Potter GD, Kreider JL, Yeates BF. 1983. Relationship between condition score, physical measurements and body fat percentage in mares. Equine Vet J. 15:371–372. 10.1111/j.2042-3306.1983.tb01826.x 6641685

[skag255-B14] Hoffmann CA et al 2020. The use of portable X-ray fluorescence spectrometry to measure apparent total tract digestibility in beef cattle and sheep. J Anim Sci. 98:skaa048. 10.1093/jas/skaa048 32052008 PMC7070150

[skag255-B15] Hudson JM , Cohen ND, Gibbs PG, Thompson JA. 2001. Feeding practices associated with colic in horses. J Am Vet Med Assoc. 219:1419–1425. 10.2460/javma.2001.219.1419 11724182

[skag255-B17] Kenward MG , Roger JH. 1997. Small sample inference for fixed effects from restricted maximum likelihood. Biometrics. 53:983–997. 10.2307/2533558 9333350

[skag255-B18] Martuzzi F , Danese T, Simoni M, Valle E, Righi F. 2024. Faecal particle size distribution in relation to forage type and digestibility in horses: preliminary results. Ital J Anim Sci. 23:1146–1152.

[skag255-B19] Microsoft Corporation. 2024. Microsoft excel (version 16.0). Microsoft.

[skag255-B20] Müller CE. 2024. Dry matter concentration, particle size distribution and sand presence in faeces from horses with and without colic. J Equine Vet Sci. 139:105126. 10.1016/j.jevs.2024.105126 38852928

[skag255-B21] NRC. 2007. Nutrient requirements of horses. 6th rev. ed. National Academies Press.

[skag255-B22] Preston L. 2016. The ultimate guide to horse feed, supplements, and nutrition. Simon and Schuster.

[skag255-B23] Retsch GmbH. 2016. SR 300 rotor beater mill. Retsch GmbH.

[skag255-B24] RStudio Team. 2023. RStudio: Integrated development environment for R (version 2023.06.2 + 561). RStudio, PBC.

[skag255-B25] Silva MDLC et al 2015. Effects of soy beverage and soy-based formula on growth, weight, and fecal moisture: experimental study in rats. J Pediatr (Rio J). 91:306–312. 10.1016/j.jped.2014.09.003 25619604

[skag255-B26] Thiex N. 2013. AOAC Official Method 990.03. Protein (crude) in animal feed: combustion method. In: Official methods of analysis of AOAC International. 19th ed. AOAC Int. 10.1093/aoac/oma.990.03

[skag255-B27] Tinker MK et al 1997. Prospective study of equine colic incidence and mortality. Equine Vet J. 29:448–453. 10.1111/j.2042-3306.1997.tb03157.x 9413717

[skag255-B28] Udén P , van Soest PJ. 1982. Comparative digestion of timothy (Phleum pratense) fibre by ruminants, equines and rabbits. Br J Nutr. 47:267–272. 10.1079/bjn19820035 6279144

[skag255-B29] van Soest PJ. 1963. Ruminant fat metabolism with particular reference to factors affecting low milk fat and feed efficiency: a review. J Dairy Sci. 46:204–216. 10.3168/jds.s0022-0302(63)89008-6

[skag255-B30] van Soest PV , Robertson JB, Lewis BA. 1991. Methods for dietary fiber, neutral detergent fiber, and nonstarch polysaccharides in relation to animal nutrition. J Dairy Sci. 74:3583–3597. 10.3168/jds.S0022-0302(91)78551-2 1660498

[skag255-B32] Weindorf DC , Chakraborty S. 2020. Portable X‐ray fluorescence spectrometry analysis of soils. Soil Science Soc of Amer J. 84:1384–1392. 10.1002/saj2.20151

