# Supplementary material for: The effects of diet on the physical characteristics of feces and fiber digestibility in healthy horses
Source: J Anim Sci. 2026 Aug 13;104:skag255. doi: 10.1093/jas/skag255 (PMC13555624; doi:10.1093/jas/skag255)
Supplement: Supplementary file 1 [file skag255_supplementary_data.docx]

**Supplementary Table S1.** Signalment, bodyweight, and body condition scores of study population.

| **Animal** | **Age** | **Sex** | **Breed** | **Day -17**  **BW** | **Day 17**  **BW** | **Day 34**  **BW** | **Day 34**  **BCS** |
| --- | --- | --- | --- | --- | --- | --- | --- |
| 1003 | 23 | Mare | QH | 1038 | 1037 | 956 | 4.5 |
| 1004 | 16 | Mare | QH | 1172 | 1196 | 1124 | 5.5 |
| 1029 | 9 | Mare | TB | 1270 | 1289 | 1215 | 5 |
| 11 | 17 | Mare | QH | 1165 | 1218 | 1110 | 5.5 |
| 35 | 18 | Mare | QH | 1186 | 1206 | 1139 | 5.5 |
| 38 | 12 | Mare | QH | 1100 | 1144 | 1095 | 6 |
| 39 | 18 | Mare | QH | 1205 | 1220 | 1146 | 5 |
| 50 | 18 | Mare | QH | 1165 | 1190 | 1092 | 6 |
| 1025 | 7 | Mare | QH | 1258 | 1286 | 1209 | 5.5 |
| 1012 | 25 | Mare | QH | 1420 | 1353 | 1460 | 5 |
| 1002 | 23 | Mare | QH | 1140 | 1101 | 1144 | 5 |
| 1018 | 26 | Gelding | QH | 1014 | 967 | 990 | 4.5 |
| 27 | 11 | Mare | QH | 1037 | 1088 | 1123 | 5.5 |
| 1011 | 5 | Gelding | QH | 1138 | 1097 | 1133 | 5.5 |
| 1014 | 23 | Gelding | QH | 1104 | 1035 | 1069 | 5 |
| 1028 | 18 | Gelding | QH | 1107 | 1054 | 1108 | 5.5 |
| 34 | 17 | Mare | QH | 1182 | 1112 | 1144 | 5 |
| 1009 | 18 | Mare | QH | 1120 | 1098 | 1113 | 5 |
| 28 | 25 | Mare | QH | 936 | 924 | 939 | 4.5 |
| 1022 | 5 | Gelding | QH | 996 | 942 | 979 | 5 |
